# Liquid Hybridization and Solid Phase Detection: A Highly Sensitive and Accurate Strategy for MicroRNA Detection in Plants and Animals

**DOI:** 10.3390/ijms17091457

**Published:** 2016-09-01

**Authors:** Fosheng Li, Lanju Mei, Cheng Zhan, Qiang Mao, Min Yao, Shenghua Wang, Lin Tang, Fang Chen

**Affiliations:** 1 Key Laboratory of Bio-resources and Eco-environment, Ministry of Education, College of Life Science, Sichuan University, Chengdu 610000, Sichuan, China; foshengli1987@163.com (F.L.); andersenli2@163.com (L.M.); 2014322040004@stu.scu.edu.cn (C.Z.); will3693@163.com (Q.M.); ym12784202921@163.com (M.Y.); shwang200@aliyun.com (S.W.); 2 Chengdu Botanical Garden, Chengdu 610083, Sichuan, China; 3 National and Local Joint Engineering Laboratory for Energy Plant Bio-Oil Production and Application, Chengdu 610000, Sichuan, China

**Keywords:** microRNA detection, liquid hybridization, digoxigenin, biotin, solid phase detection

## Abstract

MicroRNAs (miRNAs) play important roles in nearly every aspect of biology, including physiological, biochemical, developmental and pathological processes. Therefore, a highly sensitive and accurate method of detection of miRNAs has great potential in research on theory and application, such as the clinical approach to medicine, animal and plant production, as well as stress response. Here, we report a strategic method to detect miRNAs from multicellular organisms, which mainly includes liquid hybridization and solid phase detection (LHSPD); it has been verified in various species and is much more sensitive than traditional biotin-labeled Northern blots. By using this strategy and chemiluminescent detection with digoxigenin (DIG)-labeled or biotin-labeled oligonucleotide probes, as low as 0.01–0.25 fmol [for DIG-CDP Star (disodium2-chloro-5-(4-methoxyspiro{1,2-dioxetane-3,2′-(5′-chloro)tricyclo[3.3.1.13,7]decan}-4-yl)phenyl phosphate) system], 0.005–0.1 fmol (for biotin-CDP Star system), or 0.05–0.5 fmol (for biotin-luminol system) of miRNA can be detected and one-base difference can be distinguished between miRNA sequences. Moreover, LHSPD performed very well in the quantitative analysis of miRNAs, and the whole process can be completed within about 9 h. The strategy of LHSPD provides an effective solution for rapid, accurate, and sensitive detection and quantitative analysis of miRNAs in plants and animals.

## 1. Introduction

MicroRNAs (miRNAs), an abundant family of noncoding endogenous RNAs, commonly exist in plants, animals and microbes, and regulate gene expression at the post-transcriptional level by complementary base pairing and/or at the transcriptional level by DNA methylation [1,2,3]. It has been estimated that about one-third of all protein-coding genes are regulated by miRNAs [4]. miRNAs play important roles in a wide range of biological processes, including cell differentiation, development, signal transduction, protein degradation, stress responses, various diseases, and regulation of their own biogenesis [5,6,7,8,9,10]. Since the first miRNA was found in *Caenorhabditis elegans* [11,12], more than 20,000 miRNAs from 193 species have been registered in the miRNA database version 21 (http://www.mirbase.org/), but the biological functions of most miRNAs are unknown [13].

A highly sensitive and accurate detection method of miRNA expression is a critical requirement for understanding their biological functions. However, the small size of miRNAs increases the technical difficulty of their detection. Currently, the methods of miRNA detection are divided into two categories: (1) the probe-hybridization-based methods, including Northern blot, microarray, nanogold-labeling assay, and splinted ligation with radioactive labels; and (2) the amplification-based methods including real-time PCR (RT-PCR), rolling cycle amplification, invader assay, and next generation sequencing [14,15,16,17,18,19,20,21]. Among them, the most convincing and widely used analytical method is still the traditional Northern blot because of its advantages in accurately determining the expression level and size of both the small RNAs and their precursors, although sensitive RT-PCR and high-throughput microarray techniques have been developed [15,21]. The traditional Northern blot protocols include fractionating small RNAs by gel electrophoresis; transferring the separated RNA fragments onto a nylon membrane; overnight hybridization; and hours to days or even months of autoradiography [22,23,24]. However, the method is not only less sensitive, more complicated, and more time-consuming, but also expensive and unsafe to researchers and the environment.

To overcome these disadvantages, several distinct Northern blot protocols are currently used for miRNA detection, which differ in the labeling and design of the probes used to detect miRNAs. For example, locked nucleic acid (LNA)-modified oligonucleotide probes were used to enhance the efficiency of hybridization and shown to exhibit improved thermal stability when hybridized to their target molecules, but they are very expensive [24,25,26]. In contrast, digoxigenin (DIG)/biotin-labeled systems—which possess the advantages of short exposure time, better stability, inexpensive cost, and better safety—were also used to detect small RNAs recently [27,28,29,30]. Moreover, a new technology of liquid Northern hybridization, which is classified as a solution phase method compared to the solid-phase methods like the traditional Northern blots, also overcomes these shortcomings and allows quick and simple detection of miRNAs [30,31].

Here, we reported a strategical method for improving the current methods of detection of miRNAs; this method combines the liquid hybridization method with the solid phase detection method (LHSPD). Through this strategy, a highly sensitive and accurate detection of miRNAs from multicellular organisms can be completed within about 9 h, which is much more sensitive than traditional biotin-labeled Northern hybridization, and a one-base difference can be distinguished between miRNA sequences. Moreover, the hybridization signal by LHSPD has a good linear relationship with miRNA concentration, which provides the prerequisite for quantitative analysis of miRNAs. Thus, LHSPD provides an alternative strategy for convenient, reliable, and sensitive detection and quantitative analysis of miRNAs in plants and animals.

## 2. Results

**Figure 1:**
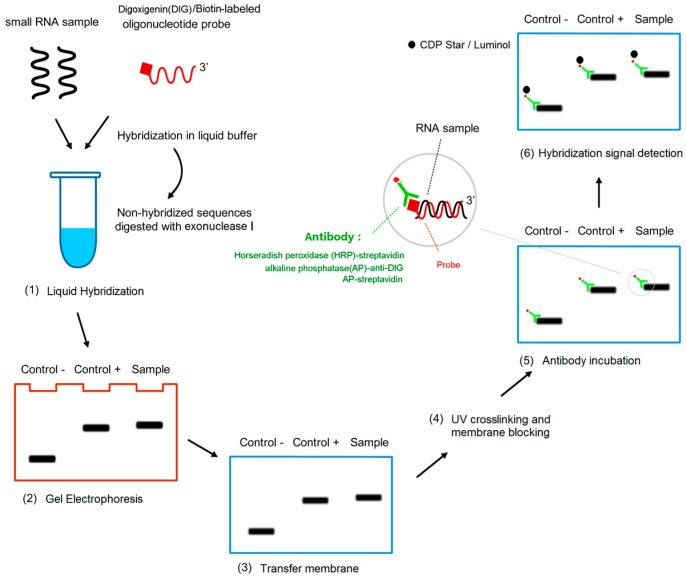
Schematic diagram of procedures of liquid hybridization and solid phase detection (LHSPD). (**1**) Liquid hybridization: The small RNA samples, hybridization buffer, and probe are mixed in a tube to make the probe hybridize with the specific RNA sequences and the non-hybridized sequences are digested by exonuclease I; (**2**) Gel electrophoresis: the products of the hybridization are separated by electrophoresis; (**3**) Transfer membrane; (**4**) UV crosslinking and membrane blocking; (**5**) Antibody incubation: alkaline phosphatase (AP)-anti-DIG antibody or AP-streptavidin or horseradish peroxidase (HRP)-streptavidin targeted the RNA-bound DIG-labeled probes or biotin-labeled probes respectively; (**6**) Hybridization signal detection: CDP-Star/luminol is used to detect the combination of antibody and target RNA.

The whole miRNA detection process is divided into two parts: liquid hybridization and detection on solid support (Figure 1).

### 2.1. Sensitivity of LHSPD

**Figure 2:**
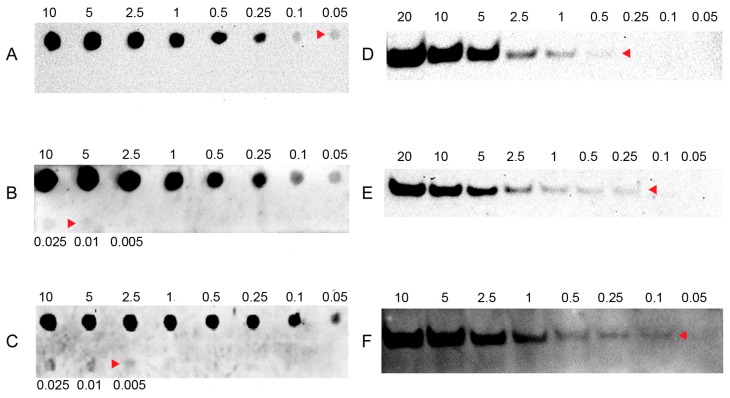
Sensitivity of LHSPD with different detection systems. (**A**,**D**) Hybridizations of different concentrations of (*Biotin*)*-miD156rk* with 0.1 pmol of *miD156* detected by the LHSPD strategy with biotin-luminol system; (**B**,**E**) Hybridizations of different concentrations of (*DIG*)*-miD156rk* with 0.1 pmol of *miD156* detected by the LHSPD strategy with DIG-CDP Star system; (**C**,**F**) Hybridizations of different concentrations of (*Biotin*)*-miD156rk* with 0.1 pmol of *miD156* detected by the LHSPD strategy with biotin-CDP Star system.

To evaluate the sensitivity of LHSPD, we firstly conducted a series of hybridizations of different concentrations of (*DIG*)*-miD156rk* or (*Biotin*)*-miD156rk* with 0.1 pmol of *miD156* at 42 °C for 60 min, followed by digestion of non-hybridized sequences with exonuclease I for 30 min. After that, dot blots and band blots were performed and detected by the LHSPD strategy with biotin-CDP Star (disodium2-chloro-5-(4-methoxyspiro{1,2-dioxetane-3,2′-(5′-chloro)tricycle [3.3.1.13,7]decan}-4-yl) phenyl phosphate) system, DIG-CDP Star system, and biotin-luminol system. The results showed that DIG-CDP Star system and biotin-CDP Star system could distinguish 0.01 fmol (10 amol) of (*DIG*)*-miD156rk* and 0.005 fmol (5 amol) of (*Biotin*)*-miD156rk* signals in dot blotting, 0.25 fmol of (*DIG*)*-miD156rk* and 0.1 fmol of *Biotin-miD156rk* signals in band blotting by CDP Star detection, respectively (Figure 2B,C,E,F). This indicates that biotin-CDP Star system is more sensitive than DIG-CDP Star system.

In chemiluminescent detection of biotin probe signal, CDP Star showed at least 5 times higher sensitivity than luminol (Figure 2A,C,D,F), and the sensitivity of luminol was 200 times higher than fluorescein isothiocyanate (FITC) (Table S1) [31].

### 2.2. Specificity and Hybridization Buffer of LHSPD

**Figure 3:**
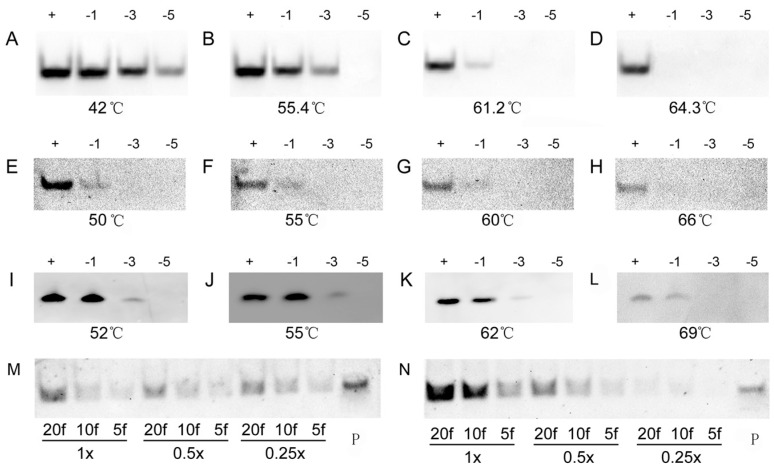
Specificity of LHSPD at different temperatures. (**A**–**D**) Hybridizations of 0.1 pmol (*DIG*)*-miD156rk* with 1 pmol *miD156s* at different temperatures by LHSPD; (**E**–**H**) Hybridizations of 0.1 pmol (*Biotin*)*-miD156rk* with 1 pmol *miD156s* at different temperatures by LHSPD; (**I**–**L**) Hybridizations of 0.1 pmol (*Biotin*)*-miD156rk* with 1 pmol *miD156s* at different temperatures by traditional Northern hybridization. *miD156* marked “+”, *miD156* with one-base mismatch marked “−1”, *miD156* with three-base mismatch marked “−3” and *miD156* with five-base mismatch marked “−5”; (**M**) Hybridization performed in 0.25×, 0.5× and 1× Exonuclease I reaction buffer (New England Biolabs, Inc., Beijing, China) with 0.1 pmol (*Biotin*)*-miD156rk*; (**N**) Hybridization performed in 0.25×, 0.5× and 1× PNE buffer with 0.1 pmol (*Biotin*)*-miD156rk*; 20f represents 20 fmol of *miD156*; 10f represents 10 fmol of *miD156*; 5f represents 5 fmol of *miD156*; P represents control containing 1 pmol of (*Biotin*)*-miD156rk*.

To further evaluate the specificity of the DIG- and biotin-labeled oligonucleotide probes in LHSPD, we performed hybridizations of 0.1 pmol (*DIG*)*-miD156rk* or (*Biotin*)*-miD156rk* with 1 pmol *miD156s* (Table 1) that included different mismatched bases at different temperatures, and detected, by the LHSPD strategy, with DIG-CDP Star system and biotin-CDP Star system, respectively. For DIG-CDP Star system, we first conducted the hybridization at 42 °C and found that the hybridization signals from all the *miD156s* were clearly visible (Figure 3A). Hence, hybridizations at higher temperature were performed and the results showed that the hybridizations could distinguish the signals from five-base difference at 55.4 °C, three-base difference at 61.2 °C, and one-base difference at 64.3 °C, which is 7 °C higher than its melting temperature (T_m_) (Figure 3B–D). For biotin-CDP Star system, we first performed the hybridization at 55 °C, mainly because it is the temperature at which the hybridization could distinguish the signal from five-base difference in DIG-CDP Star system. Surprisingly, the hybridization could distinguish the signal from three-base difference, and the signal from the one-base mismatch *miD156* was also very weakly detected (Figure 3F). Moreover, there was not much difference between the hybridization signals at 50–60 °C, and the hybridization signal from the one-base mismatch *miD156* completely disappeared at 66 °C, which is 9 °C higher than its T_m_ (Figure 3E–H). Hence, we could come to the conclusion that LHSPD could distinguish miRNAs with one-base difference, when the hybridization was performed a little higher than its T_m_. However, for comparison, the hybridizations at different temperatures were also performed by traditional Northern hybridization with biotin-CDP Star system, and the hybridization signals at 55 °C were the same as those detected by the LHSPD strategy with DIG-CDP Star system (Figure 3J). In addition, the hybridizations at 62 °C seemed to distinguish the signal from three-base difference but could not distinguish miRNAs with one-base difference, even at 69 °C (Figure 3I–L). Therefore, the specificity of LHSPD strategy was superior to the traditional Northern hybridization.

In determining the appropriate hybridization solution, we used the PNE buffer (30 mM phosphate buffer, pH = 8.0, 100 mM NaCl, 10 mM ethylenediaminetetraacetic acid (EDTA)) and exonuclease I reaction buffer (67 mM glycine-KOH, 6.7 mM MgCl_2_, 10 mM β-mercaptoethanol, pH 9.5, 25 °C) as basic solutions for screening suitable ion concentration. As Figure 3 shows, both 1× buffers, especially 1× PNE buffer, showed stronger signals (Figure 3M,N).

### 2.3. Signal Detection on Solid Support Membrane

**Figure 4:**
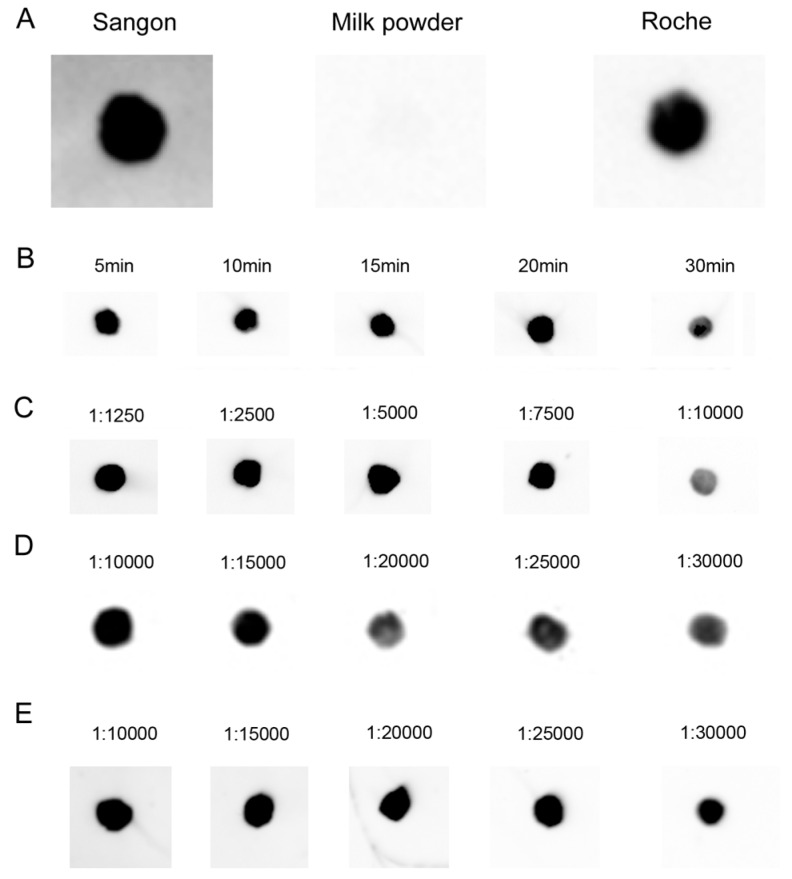
Optimization of hybridization signal detection. (**A**) 0.1 pmol biotin-labeled oligonucleotide probe was dot-blotted on a nylon membrane, followed by incubation for 1 h at room temperature, and blocked with different blocking buffers; (**B**) Blocking time of Roche blocking buffer; (**C**–**E**) Detection with 0.1 pmol DIG- and/or biotin-labeled probe and 30 min of incubation time with different diluted multiple antibodies. (**C**) Biotin-luminol (streptavidin-HRP); (**D**) DIG-CDP Star (anti-DIG-AP); (**E**) Biotin-CDP Star (streptavidin-AP).

After the DNA–RNA hybrid had been transferred onto hybridization membrane, we tested non-protein blocking buffer (Sangon Biotech Co., Ltd., Shanghai, China), TBST (TBS (Tris-buffered saline) plus 0.1%, *v*/*v*, Tween 20) containing 5% skimmed milk powder and 1× blocking buffer (Roche Applied Science, Mannheim, Germany) for screening appropriate blocking buffer. The experiments performed at room temperature showed that under the conditions of 30 min of blocking followed by washing 3 times, 10 min each, both Sangon and Roche blocking buffers give very low background (Figure 4A). What is more, the blocking was performed under the same conditions for different blocking times with Roche blocking buffer, and the differences in blocking time (5 to 20 min) had no significant impact on the results (Figure 4B).

Next, a series of dilution experiments with antibodies showed that the available working concentrations (dilutions) of antibodies were 1:1250–7500 for streptavidin-horseradish peroxidase (HRP) (the manual’s recommended working concentrations are 1:2500–1:10,000), 1:10,000–15,000 for anti-DIG antibody (the manual’s recommended working concentrations are 1:10,000–1:20,000), and 1:10,000–25,000 for streptavidin-AP (the manual’s recommended working concentrations are 1:5000–1:20,000) which is much lower than the manual’s recommended working concentrations, indicating that hybridization by the LHSPD strategy with biotin-CDP Star system needs a lower concentration of antibodies (streptavidin-AP), so it may be cheaper than other methods (Figure 4C–E.).

### 2.4. Detection of miRNAs with LHSPD

**Figure 5:**
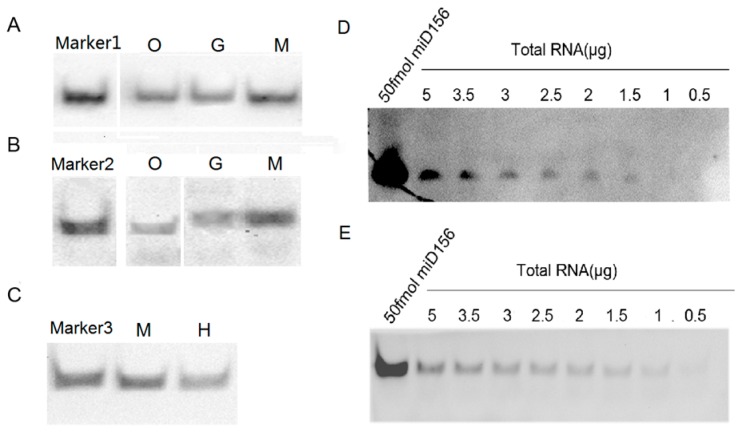
Detection of microRNAs (miRNAs) with LHSPD. (**A**) Detection by biotin-CDP Star system using 1 μg small RNA samples. Marker1: 50 fmol of *miD156*/(*Biotin*)-*miD156rk* hybrid; O: *Oryza sativa* small RNA plus 0.1 pmol (*Biotin*)*-miD156rk*; G: *Gallus domesticus* small RNA plus 0.1 pmol (*Biotin*)*-let-7rk*; M: *Mus musculus* small RNA plus 0.1 pmol (*Biotin*)*-let-7rk*; (**B**) Detection by DIG-CDP Star system using 5 μg small RNA samples. Marker2: 50 fmol of *let-7a*/(*DIG*)*-let-7rk* hybrid; O: *Oryza sativa* small RNA plus 0.1 pmol (*DIG*)*-miD156rk*; G: *Gallus domesticus* small RNA plus 0.1 pmol of (*DIG*)-*let-7rk*; M: *Mus musculus* small RNA plus 0.1 pmol of (*DIG*)*-let-7rk*; (**C**) Detection by biotin-luminol system using 5 μg small RNA samples. Marker3: 50 fmol of *let-7a*/(*Biotin*)*-let-7rk* hybrid; M: *Mus musculus* small RNA plus 0.1 pmol (*Biotin*)-*let-7rk*; H: Human blood small RNA plus 0.1 pmol (*Biotin*)*-let-7rk*; (**D**) Detection of *miR156* from *Nicotiana tabacum* total RNA samples by traditional Northern hybridization with biotin-CDP Star system: 50 fmol of *miD156* as a control and different amounts of *Nicotiana tabacum* total RNA hybridized with 0.1 pmol (*Biotin*)*-miD156rk*; (**E**) Detection of miR156 from *Nicotiana tabacum* total RNA samples by the LHSPD strategy with biotin-CDP Star system: 50 fmol of *miD156* as a control and different amounts of *Nicotiana tabacum* total RNA hybridized with 0.1 pmol (*Biotin*)*-miD156rk.*

To examine the feasibility of the protocol above for detecting miRNAs, we performed hybridizations of *miR156* and *let-7a* from small RNA samples of plants (*Oryza sativa* and *Nicotiana tabacum*) and animals (*Mus musculus*, *Gallus domesticus* and *Homo sapiens*), respectively, and their signals from the same small RNA samples were compared. The results showed that the hybridization signals detected by the biotin-CDP Star system using 1 μg small RNA samples were completely comparable with those detected by the DIG-CDP Star system using 5 μg small RNA samples, which indicated that the biotin-CDP Star system is more sensitive than the DIG-CDP Star system (Figure 5A,B). However, the hybridization signals detected by the biotin-luminol system were lower than those detected by the DIG-CDP Star system, both using 5 μg small RNA samples (Figure 5B,C); even so, they were all clearly visible, including the hybridization signal of the small RNA *let-7a* from human blood (Figure 5C). In short, our results demonstrated that *miR156* and *let-7a* had been successfully detected using the LHSPD strategy with the biotin-CDP Star system, DIG-CDP Star system, and biotin-luminol system in all small RNA samples, and that the biotin-CDP Star system is more sensitive than the DIG-CDP Star system and biotin-luminol system, which is consistent with the result indicating its sensitivity. In order to compare the LHSPD strategy and traditional Northern hybridization, we next performed detection analyses of *miR156* from different amounts of *Nicotiana tabacum* total RNA samples using the LHSPD strategy and traditional Northern hybridization with the biotin-CDP Star system. The results showed that the LHSPD strategy was more sensitive than traditional Northern hybridization, with clearer background, and could detect *miR156* from only 1 μg *Nicotiana tabacum* total RNA sample while the traditional Northern hybridization detected *miR156* from 1.5 μg *Nicotiana tabacum* total RNA sample (Figure 5D,E).

In addition, more validations of other miRNAs or from other plants, including *Jatropha* *curcas* and *Arabidopsis* *thaliana*, and other tissues as well as other animals are listed in Table S2, and the LHSPD strategy described here has been successfully used to detect novel intronic miRNAs uncovered in the rice genome, such as *miR263*, *miR557*, and *miR1188* [32].

### 2.5. Quantitative Analysis of miRNAs by LHSPD

**Figure 6:**
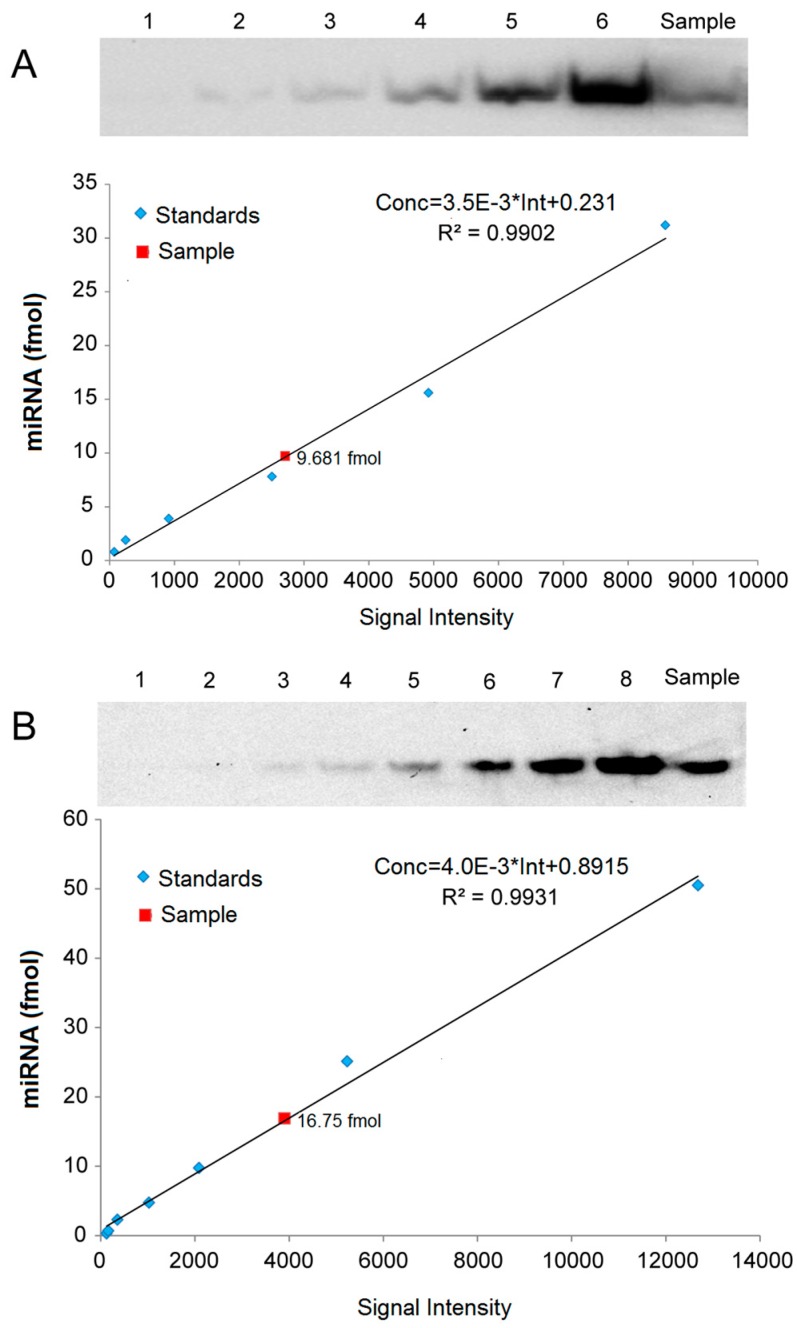

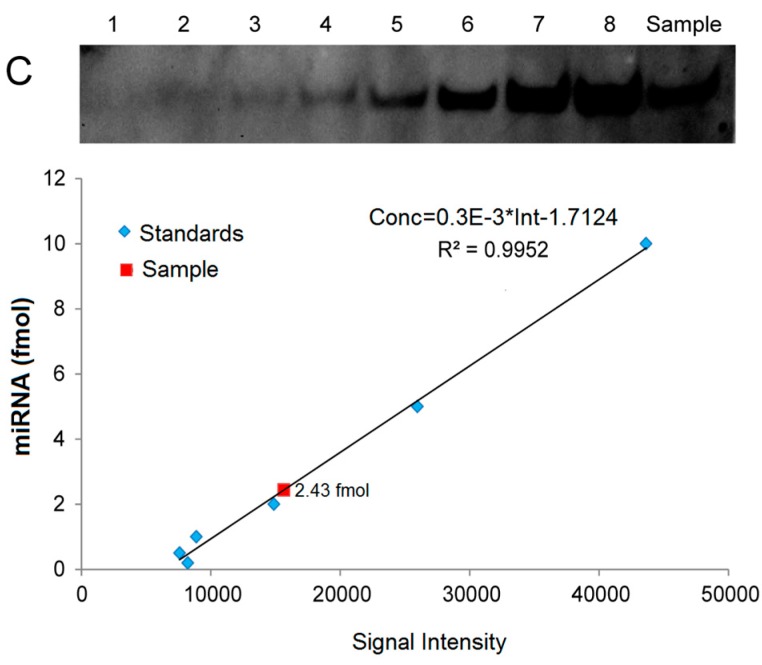
Quantitative analysis of miRNAs by LHSPD. (**A**) Quantitative analysis of *Osa-miR156* in *Oryza sativa* seedlings by LHSPD with the biotin-luminol system. **Upper**: Image of *Osa-miR156* from *Oryza sativa* seedlings; lanes 1–6 represent 0.8, 1.9, 3.9, 7.8, 15.6, and 31.2 fmol (*Biotin*)*-miD156rk*, respectively, hybridized with an excess of *Osa-miR156* to create a standard curve; lane Sample is 1 μg *Oryza sativa* small RNA sample hybridized with 0.1 pmol (*Biotin*)*-miD156rk*; **Lower**: Image is a quantification using a Bio-Rad (Irvine, CA, USA) gel imaging system; (**B**) Quantitative analysis of *let-7* in *Mus musculus* liver by LHSPD with the DIG-CDP Star system. **Upper**: Image of *let-7* from *Mus musculus* liver; lanes 1–8 represent 0.25, 0.5, 1, 2.5, 5, 10, 25, and 50 fmol (*DIG*)*-let-7rk*, respectively, hybridized with an excess of *let-7a* to create a standard curve; lane Sample is 10 μg of *Mus musculus* small RNA sample hybridized with 0.1 pmol (*DIG*)*-let-7rk*; **Lower**: Image is a quantification using a Bio-Rad gel imaging system; (**C**) Quantitative analysis of *let-7* in *Drosophila* *melanogaster* by LHSPD with the biotin-CDP Star system. **Upper**: Image of *let-7* from *Drosophila melanogaster*; lanes 1–8 represent 0.05, 0.1, 0.2, 0.5, 1, 2, 5, and 10 fmol (*Biotin*)*-let-7rk*, respectively, hybridized with an excess of *let-7a* to create a standard curve; lane Sample is 10 μg of *Drosophila* *melanogaster* small RNA sample hybridized with 0.1 pmol (*Biotin*)*-let-7rk*; **Lower**: Image is a quantification using a Bio-Rad gel imaging system.

For quantitative analysis of miRNAs, we created standard curves based on a series of hybridization signals from different concentrations of (*Biotin*)*-miD156rk* and (*DIG*)*-let-7rk* or (*Biotin*)*-let-7rk* with synthesized *Osa-miR156* and *let-7a*, respectively, and detected by LHSPD with the biotin-luminol system, DIG-CDP Star system, and biotin-CDP Star system, respectively. The results showed that the signal intensities detected by LHSPD with the biotin-luminol system, DIG-CDP Star system and biotin-CDP Star system had good linear relationships with miRNA concentrations (Conc = 3.5 × 10^−3^ × Int + 0.231, R^2^ = 0.9902, quantitation of *Osa-miR156* in *Oryza sativa* seedlings by LHSPD with the biotin-luminol system; Conc = 4.0 × 10^−3^ Int + 0.8915, R^2^ = 0.9931, quantitation of *let-7* in *Mus musculus* liver by LHSPD with the DIG-CDP system; Conc = 0.3 × 10^−3^ Int − 1.7124, R^2^ = 0.9952, quantitation of *let-7* in *Drosophila* *melanogaster* by LHSPD with the biotin-CDP Star system) (Figure 6). By this quantitative analysis method, 9.681 fmol of *Osa-miR156* was detected from 1 μg rice small RNA sample by LHSPD with the biotin-luminol system, and 16.75 fmol and 2.43 fmol of *let-7* were detected from 10 μg *Mus musculus* and *Drosophila* *melanogaster* small RNA samples by LHSPD with DIG-CDP Star system and Biotin-CDP Star system, respectively (Figure 6).

## 3. Discussion

In this paper, we combined liquid hybridization with solid-phase detection and developed a highly sensitive and accurate method for the detection of miRNAs in plants and animals. The liquid hybridization consists of RNA hybridization with the DIG- or biotin-labeled probe, exonuclease I treatment to digest non-hybridized single-strand nucleic acids, and polyacrylamide gel electrophoresis (PAGE), while the solid-phase detection includes transfer of the hybrids and detection of the hybridization signal.

Liquid hybridization based on FITC, a small molecular fluorescent substance, is characterized by rapidness and accuracy [31], because the rate of molecular motion and collision frequency in solution are greater than on a solid support membrane. Furthermore, hybridization temperature can be effectively controlled, thus hybridization stringency can be precisely controlled. Similar to FITC, DIG and biotin are small molecular substances (Table S3), and the DIG- or biotin-labeled sequence probes have similar behavior in liquid hybridization and PAGE, and thus can also be used to detect miRNAs and other types of small RNAs. However, due to their non-fluorescence, hybrids from DIG- or biotin-labeled probes cannot be measured directly. This makes LHSPD take more time than FITC for detecting the hybridization signal [31]. However, its hairpin property makes it possible to detect miRNAs more sensitively by the chemiluminol enzyme immunoassay. 

This strategic method can determine as low as 0.005 fmol miRNA signal for dot blotting and 0.1 fmol for band blotting by the biotin-CDP Star system, which is slightly more sensitive than the DIG-CDP Star system. This might be because the association of biotin with streptavidin or avidin is the strongest known noncovalent protein–ligand interaction (K_a_ ≈ 2.5 × 10^13^ M^−1^), which might be greater than the interaction between DIG and the anti-DIG antibody [33], and the better conjunction could improve the sensitivity during antibody incubation. The above studies used short single-strand DNA instead of miRNAs as controls. Since the structure of DNA–RNA is thermodynamically more stable than DNA–DNA, hybrids of DNA–DNA could better reflect the sensitivity of LHSPD [34]. Although its sensitivity is lower than the RT-PCR method (tens of copies per microliter) and Nilsen’s ^32^P-labeled splinted ligation method (since ^32^P could improve the sensitivity of Northern blot, 0.01–0.02 fmol miRNAs) [35,36], it could avoid the risk to the health of users and detect low expression of miRNAs, including intronic miRNAs, such as *miR263* and *miR557* in rice [32]. More importantly, LHSPD was more sensitive than traditional Northern hybridization, as indicated by the fact that the *miR156* signal can be effectively detected from a small amount (1 μg) of *Nicotiana tabacum* total RNA sample by LHSPD.

The specificity of the Northern blot varies among miRNAs since short DNA–RNA hybrids show melting temperatures and binding dynamics that vary significantly with different situations [37]. This strategic method can distinguish single-base mismatches when the hybridization is performed a little higher than the T_m_ and is superior to the traditional Northern hybridization. It might be because the rate of molecular motion and collision frequency on a solid support membrane is lower than in solution, leading to hybridization with less stringency. In addition, LHSPD can be performed in quantitative analysis of miRNAs, and the whole process just needs about 9 h, far less than traditional Northern hybridization (2 days or more). Therefore, LHSPD provides an alternative effective solution for rapid, sensitive, and accurate detection and quantitative analysis of small RNAs. It not only enhances research, but also helps reduce overall costs and promotes more environmentally friendly laboratory practices. 

## 4. Experimental Section

### 4.1. Plant and Animal Materials

Plants of *Oryza sativa*, *Arabidopsis thaliana* (Columbia ecotype), *Nicotiana tabacum* and *Jatropha curcas* were grown in the greenhouse of the Laboratory of Bio-resources and Eco-environment, College of Life Science, Sichuan University (Chengdu, China), at 25 °C under a 16/8-h-light/dark photoperiod.

*Drosophila melanogaster* stocks were maintained on a standard cornmeal–yeast–agar medium at 25 °C. *Mus musculus* were obtained from the Experimental Animal Center of Sichuan University (Chengdu, China), and *Gallus domesticus* from Sichuan Chicken Breeder Farm. All animal experiments were approved by the Animal Experimental Ethical Committee of Sichuan University, Chengdu, China, Permit number: SCXK (chuan) 2013-026. Human blood samples were collected into disposable vacuum blood collection tubes from healthy adults (with the approval by the Ethics Committee of West China Center of Medical Sciences, Sichuan University on 5 March 2010 (SCU20100196494)) and immediately used to extract RNAs. 

### 4.2. Synthesis of Oligonucleotides and Hybridization Probes

Hybridization probes and other series of oligonucleotides (Table 1) used for miRNA detection were designed based on *Osa-miR156* and *let-7a* sequences (found at http://www.mirbase.org/) [13], which are highly conserved over a wide range of plant and animal species; they were synthesized by TaKaRa Biotechnology (Dalian, China) Co., Ltd. according to the method described previously [31,38].

### 4.3. Preparation of Small RNAs

The small RNAs for hybridization were enriched from the total RNAs. Total RNAs were isolated by Trizol reagent (Invitrogen, Shanghai, China) according to the instruction protocol, and the enrichment of small RNAs was conducted according to the method of Chun et al. [39]. After RNA precipitation by equal volume of isopropanol, the pellet was resuspended in 400 μL RNase-free water and then added to 50 μL of 50% polyethylene glycol (PEG) 8000 and 50 μL of 5 M NaCl, mixed and incubated at −20 °C for at least 30 min, followed by centrifugation at 14000 × *g* at 4 °C for 10 min. Then the PEG-NaCl precipitation procedure was repeated once. The supernatant was transferred to a new microcentrifuge tube and 1/10 volume of 1 M MgCl_2_ and 2.5 volume of absolute ethanol were added. After incubation for at least 30 min at −20 °C, the mix was centrifuged for 10 min at 14,000 × *g* at 4 °C. The pellet was washed with 75% absolute ethanol, dried at room temperature, dissolved in RNase-free water, and stored at −70 °C.

### 4.4. Protocol for miRNA Detection by LHSPD

#### 4.4.1. Liquid Hybridization

Liquid hybridization was conducted according to Wang’s method [31]. A certain amount (i.e., 1 μg) of small RNA, synthesized probe (i.e., 0.1 pmol), hybridization buffer (30 mM sodium phosphate buffer at pH 8.0, 0.3 M of NaCl, 10 mM of EDTA) were added into a RNase-free tube, and RNase-free water was added up to 17 μL. After mixing thoroughly, the reaction mixture was heated to 94 °C for 5 min and then quickly incubated for 60 min at 42–70 °C (the recommended hybridization temperature is about 10 °C below the T_m_, increase of temperature will improve the specificity). After that, non-hybridized single-stranded DNA, including the probe, was digested with 1 U exonuclease I (New England BioLabs, Inc., M0293, Beijing, China) in the same tube according to the instruction protocol for 30 min at 37 °C.

#### 4.4.2. Gel Electrophoresis

1-mm-thick non-denaturing 12% Tris/borate/EDTA (TBE)-polyacrylamide gel was prepared according to standard protocol. After gel polymerization, pre-electrophoresis was performed at 200 V for 30 min. Then, the digested hybridization products were loaded without denaturation after addition of 2 μL of 10× gel loading buffer (30 mM EDTA, 0.25% (*w*/*v*) xylene cyanol FF, 0.25% (*w*/*v*) bromophenol blue, 50% (*v*/*v*) glycerol) and the gel was run at 10–13 V/cm for 1–2 h.

#### 4.4.3. Transfer Membrane 

After electrophoresis completion, the gel was rinsed once with transfer buffer (0.5× TBE buffer) and placed onto the anode (bottom electrode) in the “sandwich-like” order, that is, 3-mm filter paper (soaked in transfer buffer)–gel–nylon membrane (presoaked in RNase-free H_2_O)–3-mm filter paper. Transfer membrane was conducted at a fixed voltage of 20 V for 1 h at 4 °C in the Mini Trans-Blot^®^ electrophoretic transfer cell (Bio-Rad).

#### 4.4.4. UV Crosslinking and Membrane Blocking

The membrane was placed on the filter paper and soaked with 1× TBE buffer, with the surface containing nucleic acid hybrids facing up. The membrane was exposed to ultraviolet irradiation (254 nm), 15–20 KJ/cm^2^ for 5–9 min at room temperature. The membrane was then blocked in 1× Roche blocking buffer (Roche Applied Science, Cat. No. 11096176001), which was diluted by maleic acid buffer (100 mM maleic acid, 150 mM NaCl, pH 7.5) for 5–20 min with gentle shaking at room temperature.

#### 4.4.5. Antibody Incubation

Antibodies (streptavidin-HRP (Invitrogen, Cat. No. 43-4323), streptavidin-AP (Roche Applied Science, Cat. No. 11089161001) or anti-digoxigenin-AP (Roche Applied Science, Cat. No. 11093274910)) were diluted in 1× Roche blocking buffer to the dilutions of 1:1250–10,000 for streptavidin-HRP, 1:10,000–30,000 for anti-DIG antibody and streptavidin-AP. The membrane was rinsed with wash buffer (100 mM maleic acid, 150 mM NaCl, 0.3% Tween-20, pH 7.5) and incubated in dilute solution of antibody (50–100 mL per cm^2^ of membrane) for 30 min with gentle shaking, followed by washing with wash buffer 3 times, each wash for 10 min at room temperature.

#### 4.4.6. Hybridization Signal Detection 

For DIG-labeled probe signal, the hybridization signal was detected with CDP Star (Roche) as the reaction substrate according to the manufacturer’s instructions. The membrane (nucleic acid side facing up) was immersed in detection buffer for 3–5 min and placed on saran wrap. CDP Star was added onto the membrane (1 mL per 100 cm^2^), immediately followed by covering the membrane with another saran wrap (without air bubbles between CDP Star spreads and the membrane) and incubating for 5 min at room temperature. Incubating the damp membrane for an additional 30 min at 37 °C can enhance the luminescence signal. Finally, the luminescence signal was detected by ChemiDoc XRS (Bio-Rad) equipped with a 560DF50nm, 62 mm emission filter and a signal accumulation mode for 20 min of the exposure time. The figures with the maximal signal were selected.

For biotin-labeled probe signal, the hybridization signal was detected with luminol and CDP Star (Roche), respectively, as reaction substrates according to the manufacturer’s instructions. For luminol chemiluminescent detection, the detect buffers A and B (in Horseradish Peroxidase Detection Kit; Roche Applied Science) were mixed in a ratio of 1:1. The membrane (nucleic acid side up) was placed on saran wrap and immersed in 1 mL of the buffer mixture (1 mL per 100 cm^2^). The membrane was then incubated in the dark for 30 min at room temperature. Finally, the hybridization signal (luminescence) was detected by ChemiDoc XRS (Bio-Rad) equipped with a 560DF50nm, 62 mm emission filter and a signal accumulation mode for 20 min of the exposure time, and the figures with the maximal signal were selected. As for CDP Star chemiluminescent detection, the operation process was the same as for the DIG-labeled probe signal.

### 4.5. Traditional Northern Hybridization Strategy

#### 4.5.1. Preparation of Denaturing PAGE and Gel Electrophoresis

1-mm-thick 15% denaturing gel was prepared using 1.5 mL 10× TBE, 3.75 mL 30% acrylamide/bisacrylamide solution (29:1, Bio-Rad, Cat. No. 161-0156), 4.8 g urea, 1.45 mL ddH_2_O, 60 μL ammonium persulfate and 5 μL tetramethylethylenediamine (TEMED). After gel polymerization, pre-electrophoresis was performed at 200 V for 30 min. Then, the RNA samples were mixed with equal volumes of 2× loading buffer (95% formamide, 0.025% xylene cyanol FF, 0.025% bromophenol blue, 18 mM EDTA, 0.025% sodium dodecyl sulfate (SDS)), loaded onto the gel after heating at 95 °C for 7 min, and immediately put on ice for 5 min, after which the gel was run at 10–13 V/cm for 1–2 h.

#### 4.5.2. Transfer Membrane and UV Crosslinking

After electrophoresis completion, the gel was rinsed once with transfer buffer (0.5× TBE buffer) and placed onto the anode (bottom electrode) in the “sandwich-like” order, that is, 3-mm filter paper (soaked in transfer buffer)–gel–nylon membrane (presoaked in RNase-free H_2_O)–3-mm filter paper. Transfer membrane was conducted at a fixed voltage of 20 V for 1 h at 4 °C in the Mini Trans-Blot^®^ electrophoretic transfer cell (Bio-Rad, Inc.).

The membrane was placed on the filter paper and soaked with 1× TBE buffer, with the surface containing nucleic acid hybrids facing up. The membrane was then exposed to ultraviolet irradiation (254 nm), 15–20 KJ/cm^2^ for 9 min at room temperature, and blocked in 1× Roche blocking buffer for 30 min with gentle shaking at room temperature.

#### 4.5.3. Prehybridization and Hybridization

After completion of UV crosslinking, the membrane with RNA-side-up was rolled and inserted into the hybridization tube. Ten milliliters of hybridization buffer (2.5 mL of 20× saline sodium citrate (SSC), 0.2 mL of Na_2_HPO_4_ (1 M, pH 7.2), 7 mL of 10% SDS, 0.3 mL of 50× Denhardts, 20 mg of salmon sperm DNA) was added and prehybridized at 50 °C for 1 h in a hybridization oven.

A volume of 1.8 μmol of the probe was denatured at 95 °C for 7 min immediately following ice bath for 5 min and added into the hybridization tube and hybridized at 42–70 °C with slow rotation for 4 h. 

#### 4.5.4. Washing Membrane

After discarding the hybridization buffer from the tube, the membrane was washed twice with the buffer 1 (2× SSC, 0.1% SDS) at 25 °C for 20 min, and then twice with buffer 2 (0.1× SSC, 0.1% SDS) at 25 °C for 10 min.

#### 4.5.5. Antibody Incubation and Signal Detection

The process from antibody incubation to hybridization detection was conducted as described above.

### 4.6. Quantitative Analysis of miRNA in Small RNA Samples

Quantitation analysis of miRNAs in small RNA samples was performed according to the method of Wang et al. [31]. After the reaction completion for signal detection, the membrane was placed on a citaBlue conversion screen in a ChemiDoc XRS System camera (Bio-Rad) equipped with a 560DF50nm, 62 mm emission filter and auto-exposure. The imaging data were analyzed with Quantity One 1-D Analysis Software (Bio-Rad).

## Figures and Tables

**Table 1:** MicroRNAs (miRNAs) and derived Oligo-miD and Oligo-miD probe sequences used in liquid hybridization and solid phase detection (LHSPD).

Names	Sequence (5′–3′)	T_m_ (°C)	*M*w
*Osa-miR156*	UGA CAG AAG AGA GUG AGC AC	57.3	6279
(*Biotin*)*-miD156rk*	(Biotin)-A CGT GCT CAC TCT CTT CTG TCA	60.5	6587+
(*DIG*)*-miD156rk*	(DIG)-A CGT GCT CAC TCT CTT CTG TCA	60.5	6587+
*miD156*	TGA CAG AAG AGA GTG AGC AC	57.3	6201+
*miD156*(*−1*)	TGA gAG AAG AGA GTG AGC AC	55.4	6241
*miD156*(*−3*)	aGA CAG gAG gGA GTG AGC AC	57.4	6242
*miD156*(*−5*)	aGA CAG gAG tGA GTc AGC gC	59.5	6193+
*let-7a*	UGA GGU AGU AGG UUG UAU AGU U	46.1	7244
(*Biotin*)*-let-7rk*	(Biotin)-ACA ACT ATA CAA CCT ACT ACC TCA	52.6	7166+
(*DIG*)*-let-7rk*	(DIG)-ACA ACT ATA CAA CCT ACT ACC TCA	52.6	7166+

rk represents probe sequence; (*−*1) represents one-base mismatch sequence; (*−*3) represents three-base mismatch sequence; (*−*5) represents five-base mismatch sequence. Probes of *Osa-miR156* and *let-7a* include two additional nucleotides that do not match the target miRNA at the 5′ end [38]. DIG: digoxigenin; T_m_: melting temperature; *M*w: molecular weight.

## Supplementary Materials

Supplementary materials can be found at www.mdpi.com/1422-0067/17/9/1457/s1.
