# Supplementary material for: Liquid Hybridization and Solid Phase Detection: A Highly Sensitive and Accurate Strategy for MicroRNA Detection in Plants and Animals
Source: Int J Mol Sci. 2016 Sep 1;17(9):1457. doi: 10.3390/ijms17091457 (PMC5037736; doi:10.3390/ijms17091457)
Supplement: Supplementary file 1 [file ijms-17-01457-s001.pdf]

Supplementary Materials: Liquid Hybridization and Solid Phase Detection: A Highly Sensitive and Accurate Strategy for MicroRNA Detection in Plants and Animals

Fosheng Li, Lanju Mei, Cheng Zhan, Qiang Mao, Min Yao, Shenghua Wang, Lin Tang and Fang Chen

Table S1. Difference in detections between digoxigenin DIG/Biotin/fluorescein isothiocyanate (FITC) systems.

		DIG	Biotin		FITC [31]
Detection buffer (pH 9.5)		Yes (3–5 min)	No	No	No
Substrate for detection		CDP Star [a]	CDP Star	Luminol [b]	FITC
Enzyme used for crosslinking		Alkaline phosphatase	Alkaline phosphatase	Horseradish peroxidase	No
Incubation time for detection		5 min	5 min	5 min	No
Exposure time		5–20 min	5–20 min	5–20 min	Instantly
Duration of luminescence		24–48h	24–48 h	6 h	Seconds–minutes
Supporting multiple exposure		Yes	Yes	Yes	Yes (adding other color labels)
Identification of single-base differences		Yes	Yes	Yes	Yes
Sensitivity	Dot blot	0.01 fmol	0.005 fmol	0.05 fmol	0.01 pmol
	Band blot	0.25 fmol	0.1 fmol	0.5 fmol	0.1 pmol

[a] CDP Star: disodium 3-[4-methoxyspiro{1,2-dioxetane-3,2′-[5′-chloro]tricyclo[3.3.1.13,7]decan}-4-yl] phenyl phosphate; [b] Luminol: 5-amino-2,3-dihydro-1,4-phthalazinedione.

Table S2. MicroRNA (miRNA) detection from different organisms by liquid hybridization and solid phase detection (LSHPD) strategy.

miRNAs	Species (tissues)	Sequences (5′→3′)	Results
Osa-miR156	*Oryza sativa*-stem	UGA CAG AAG AGA GUG AGC AC	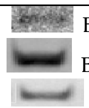 Biotin-Luminol<br>Biotin-CDP Star<br>DIG-CDP Star
Osa-miR156	*Oryza sativa*-roots	UGA CAG AAG AGA GUG AGC AC	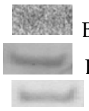 Biotin-Luminol<br>Biotin-CDP Star<br>DIG-CDP Star
Osa-miR156	*Oryza sativa*-leaves	UGA CAG AAG AGA GUG AGC AC	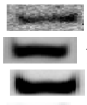 Biotin-Luminol<br>Biotin-CDP Star<br>DIG-CDP Star
miR263	*Oryza sativa*-leaves	GUG GGU CUA AGG ACU AUA UUA ACC	Biotin-CDP Star
miR557	*Oryza sativa*-leaves	AUU UGU UGU AUU AGG GAA UGU CUC	Biotin-CDP Star
miR1188	*Oryza sativa*-leaves	UGG AUG UGA CAU ACU CUA GUA	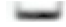 Biotin-CDP Star
miR156	*Jatropha curcas*-leaves	UGA CAG AAG AGA GUG AGC AC	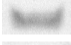 Biotin-CDP Star
miR156	*Arabidopsis thaliana*-leaves	UGA CAG AAG AGA GUG AGC AC	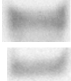 Biotin-CDP Star
let-7a	*Homo sapiens*-blood	UGA GGU AGU AGG UUG UAU AGU U	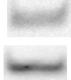 Biotin-Luminol
let-7a	*Mus musculus*-muscle	UGA GGU AGU AGG UUG UAU AGU U	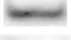 Biotin-CDP Star
let-7a	*Gallus domesticus*-blood	UGA GGU AGU AGG UUG UAU AGU U	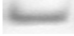 Biotin-CDP Star

miR263 and *miR557* are low-abundance intronic miRNAs and *miR1188* is an abundant intronic miRNA in *Oryza sativa* [32].

Table S3. Some properties of FITC, biotin and digoxigenin.

Name	Molecular Formula	Molecular Weight	Chemical Structure
Biotin	$C_{10}H_{16}N_2O_3S$	244.31	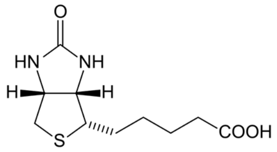
Digoxigenin (DIG)	$C_{23}H_{34}O_5$	390.51	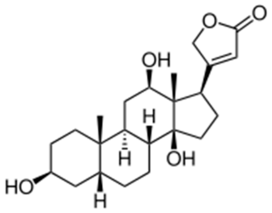
Fluorescein isothiocyanate (FITC)	$C_{21}H_{11}NO_5S$	389.382	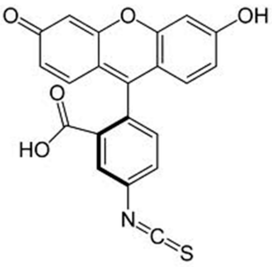
